# Indigenous injury outcomes: life satisfaction among injured Māori in New Zealand three months after injury

**DOI:** 10.1186/1477-7525-11-120

**Published:** 2013-07-17

**Authors:** Emma H Wyeth, Sarah Derrett, Brendan Hokowhitu, Ari Samaranayaka

**Affiliations:** 1 Te Roopū Rakahau Hauora Māori a Kāi Tahu (Ngāi Tahu Māori Health Research Unit), Department of Preventive and Social Medicine, Dunedin School of Medicine, University of Otago, Dunedin, New Zealand; 2 Injury Prevention Research Unit, Department of Preventive and Social Medicine, Dunedin School of Medicine, University of Otago, Dunedin, New Zealand; 3 Faculty of Native Studies, University of Alberta, Edmonton, Alberta, Canada

**Keywords:** Māori, Indigenous, Injury, Outcomes, Life satisfaction

## Abstract

**Background:**

Māori, the indigenous population of New Zealand, experience numerous and consistent health disparities when compared to non-Māori. Injury is no exception, yet there is a paucity of published literature that examines outcomes following a wide variety of injury types and severities for this population. This paper aims to identify pre-injury and injury-related predictors of life satisfaction three months after injury for a group of injured Māori.

**Methods:**

The Māori sample (n = 566) were all participants in the Prospective Outcomes of Injury Study (POIS). POIS is a longitudinal study of 2856 injured New Zealanders aged 18–64 years who were on an injury entitlement claims’ register with New Zealand’s no-fault compensation insurer. The well-known Te Whare Tapa Whā model of overall health and well-being was used to help inform the selection of post-injury life satisfaction predictor variables. Multivariable analyses were used to examine the relationships between potential predictors and life satisfaction.

**Results:**

Of the 566 Māori participants, post-injury life satisfaction data was available for 563 (99%) participants. Of these, 71% reported satisfaction with life three months after injury (compared to 93% pre-injury). Those with a higher injury severity score, not satisfied with pre-injury social relationships or poor self-efficacy pre-injury were less likely to be satisfied with life three months after injury.

**Conclusions:**

The large majority of Māori participants reported being satisfied with life three months after injury; however, nearly a third did not. This suggests that further research investigating outcomes after injury for Māori, and predictors of these, is necessary. Results show that healthcare providers could perhaps put greater effort into working alongside injured Māori who have more severe injuries, report poor self-efficacy and were not satisfied with their pre-injury social relationships to ensure increased likelihood of satisfaction with life soon after injury.

## Background

Like most indigenous populations throughout the world, Māori, the indigenous peoples of New Zealand, have marked and consistent health disparities as compared to non-indigenous New Zealanders [1-3]. Injury is no exception. Compared to their non-Māori peers, Māori aged 15–64 years have higher rates of hospitalisation (1788.0 per 100,000 compared to 1104.5 per 100,000; RR 1.62, 95% CI 1.59-1.65) and mortality (42.8 per 100,000 compared to 18.7 per 100,000; RR 2.29. 95% CI 2.05-2.56) due to unintentional injury [4]. Injuries are also responsible for disability in 31% of disabled Māori adults (aged 15–64 years) [5]. Despite such large disparities between Māori and non-Māori, there is little published research investigating outcomes after injury among Māori. The literature that does exist has tended to focus on specific injury types or outcomes, such as serious injuries or fatalities [6-10]. While this is useful, information about outcomes following a wider range of injury types and severities would contribute to identifying opportunities to reduce such disparities.

The Accident Compensation Corporation (ACC) is responsible for New Zealand’s no-fault injury compensation scheme and provides support for treatment, rehabilitation and compensation for lost earnings. Despite the high rates of injury disability and mortality outlined above, Māori have lower rates of access to ACC services than non-Māori (this reflects the more general situation where indigenous peoples and ethnic minorities often have lower access and utilisation rates of healthcare services [11]). While Māori represent 14.6% of the total New Zealand population, only 11.6% of total ACC claims between 2004 and 2009 were from Māori [12]. Māori also have disproportionately lower claim rates for social and vocational rehabilitation services, with just 6.6% of such claims being from Māori. It is hypothesised that such disparities may arise from Māori not accessing the ACC scheme as readily as non-Māori for ‘minor’ injuries, and that Māori may encounter barriers to such access because of a lack of information about their ACC entitlements [12].

In New Zealand, a study of outcomes following injury (the Prospective Outcomes of Injury Study; POIS) is underway. POIS is a large longitudinal study of injured New Zealanders and has gathered information about a large number of variables and outcomes using comprehensive structured questionnaires administered mainly via telephone interviews from late-2007; a small proportion (11%) completed postal questionnaires as a preferred option [13]. Considerable effort went into ensuring POIS would be able to make meaningful contributions to knowledge of Māori injury outcomes. Such approaches included: translation of the questionnaire into te reo Māori (the Māori language), appointing interviewers fluent in te reo Māori, recruiting sufficient numbers of Māori participants to allow Māori-specific quantitative analyses, and inclusion of a Māori-specific qualitative component [13-15]. Building on this foundation, the research team planned for this first dedicated analysis of quantitative data from Māori POIS participants to be carried out within a relevant and appropriate framework. Such efforts reflect the growing importance of incorporating indigenous epistemologies into the design of health-related research projects [16]. We have intentionally not compared findings between Māori and non-Māori in this paper. Focussing specifically on outcomes for Māori provides greater insight into areas that require further attention for this particular group.

In an effort to conceptualise Māori health perspectives and beliefs, many Māori health models have been developed, particularly since the ‘Māori renaissance’ of the 1970s. Te Whare Tapa Whā (literally, a four-sided house) is one of the most widely-acknowledged of these models [17]. Te Whare Tapa Whā was conceived in alignment with the belief of many Māori that health and well-being is not related to physical or biological factors alone, but strongly to spiritual and emotional factors too. The model consists of four dimensions, which are likened to the four walls of a house (whare), each being required to achieve and maintain overall strength and balance. These are: taha whānau (the family dimension), taha wairua (the spiritual dimension), taha tinana (the physical dimension), and taha hinengaro (the mental dimension). All dimensions interact, and are necessary, to contribute to a holistic concept of overall health and well-being [17,18].

This present research is unique in that Te Whare Tapa Whā’s concept for overall health and well-being was used to inform the selection of the current POIS outcome of interest – life satisfaction in the sub-acute injury phase. POIS questionnaires were not conceived to measure or reflect specific dimensions of Te Whare Tapa Whā. However, because POIS questionnaires were designed to include a wide range of socio-demographic, health, disability and injury-related variables, by their nature many variables are connected to one, or more, dimensions of Te Whare Tapa Whā.

The purpose of this paper is to identify which pre-injury variables, including those relevant to Te Whare Tapa Whā and some additional socio-demographic factors, relate to overall life satisfaction in the sub-acute post-injury period. This paper has two aims: 1) to present some baseline characteristics of the total Māori cohort of POIS; and 2) to investigate potential predictors (identified with reference to Te Whare Tapa Whā) of post-injury life satisfaction for this cohort.

## Methods

This paper uses data from POIS (n = 2856), which recruited participants via the Accident Compensation Corporation of New Zealand. The recruitment process and cohort characteristics for this study have been described in detail elsewhere [13,14], however, a brief overview is provided below.

Eligible POIS participants were injured New Zealanders, aged 18–64 years inclusive, living in one of five study regions, who had sustained an injury between June 2007 and May 2009 and were referred to the ACC and subsequently placed on ACC’s entitlement claims’ register. This register comprises injured people who are likely to require more than treatment-only assistance, regardless of how they were injured, as assessed by ACC at the time of the injury claim. Assistance can include home-help, compensation for loss of earnings and travel assistance [19]. Those injured as a result of self-harm or sexual assault were excluded from POIS.

The first POIS interviews were carried out between December 2007 and August 2009 (on average, three months after injury) and collected pre-injury and sub-acute post-injury data from the 2856 participants. This paper focuses on the data of those who self-reported Māori ethnicity [13].

This study received ethical approval from the New Zealand Health and Disability Multi-region Ethics Committee (MEC/07/07/093).

### Descriptive characteristics of Māori POIS cohort

This is the first POIS paper that specifically focuses on analyses of data provided by Māori participants of POIS. Therefore, a range of baseline pre-injury characteristics is also presented in this paper.

Participants were asked to report their gender, age, ethnicity, and if they knew the name(s) of their iwi (tribe) using questions from the New Zealand Census [20]. All participants were asked to report which ethnic group(s) they identified with. Region of residence within New Zealand at the time of injury was obtained from ACC records.

Level of highest educational qualification was also obtained using questions from the New Zealand Census [20]. Education responses were grouped as ‘no secondary school’, ‘secondary school’ and ‘post-secondary school’ (that took ≥3 months to complete) qualifications. People were also asked whether they were working for pay at the time of their injury and grouped accordingly to ‘full-time (≥30 hours per week)’ or ‘part-time (<30 hours per week)’ or ‘not in paid employment’ [21]. Total personal income in the 12 months prior to injury was also reported in pre-tax New Zealand dollars (i.e. ≤$15,000; $15,001-$30,000; $30,001-$50,000; $50,001-$70,000; ≥$70,001;and, undisclosed).

Self-reported rating of overall health before injury was also recorded on a five-point scale of ‘excellent’, ‘very good’, ‘good’, ‘fair’ or ‘poor’ and grouped as ‘good/very good/excellent’ and ‘fair/poor’ [22]. Pre-injury chronic illnessess were reported using questions modified from the New Zealand Health Survey 2006/2007 [23]. Participants were asked if they had ever been told by a doctor before their injury that they had any of 22 specified chronic illnesses or diseases (such as asthma, depression, diabetes or cancer) that had lasted, or was expected to last, for more than six months.

Injury severity was measured using a New Injury Severity Score (NISS) derived for each participant from injury diagnosis data provided, with participants’ consent, by ACC [24]. Injury severity scores were grouped: NISS 1–3 (least severe), NISS 4–6 (middle severity), and NISS >6 (most severe). A more detailed description of the derivation of these scores for the POIS cohort has been previously published [25].

### Outcome variable

Life satisfaction was chosen as the outcome variable of interest as it seemed most-closely aligned with the overall health and well-being concept encapsulated by Te Whare Tapa Whā. Life satisfaction was assessed by asking participants how satisfied they were with their “life as a whole” at the time of the interview [26]. Response options were ‘completely satisfied’, ‘mostly satisfied’, ‘neither satisfied nor dissatisfied’, ‘mostly dissatisfied’, ‘completely dissatisfied’ or ‘don’t know’. For the analyses, the first two responses were categorised as ‘satisfied’, the following three as ‘not satisfied’ and ‘don’t know’ responses as ‘missing’.

### Explanatory variables

Potential pre-injury and injury-related explanatory variables were obtained at the first POIS interview (approximately three months after injury) and grouped into Te Whare Tapa Whā dimensions and socio-demographic characteristics. As discussed previously, Te Whare Tapa Whā, in conjunction with existing injury and life satisfaction literature, was used to help inform key variables that might be important potential predictors of life satisfaction for Māori following injury. The large majority of the questionnaire was able to be categorised into one of the four Te Whare Tapa Whā dimensions. For practical reasons, three authors (EW, BH and SD) met and discussed which variables were hypothesised to be most important to investigate their relationship with the life satisfaction soon after injury.

#### Te Whare Tapa Whā characteristics

##### Whānau (family) dimension

Although whānau is commonly translated as ‘family’, in the context of Te Whare Tapa Whā this concept is often associated with social relationships more generally [17]. Participants were asked to report their overall satisfaction with social relationships (such as the quality and frequency of relationships and contact with their partner, relatives and friends) rated as ‘completely satisfied’, ‘mostly satisfied’, ‘neither satisfied or dissatisfied’, ‘mostly dissatisfied’, or ‘completely dissatisfied’. The first two responses were categorised as ‘satisfied’; the remaining three as ‘not satisfied’. Whether family/whānau played a ‘very large’, ‘large’, ‘small’ or ‘very small’ part in their lives before their injury was also reported and grouped as ‘very large/large’ and ‘small/very small’ [21].

##### Wairua (spiritual) dimension

Wairua is an esoteric notion that has no readily translatable English equivalent. For instance, both animate and inanimate entities can have wairua. In light of this it has often been described as ‘soul’ and/or quintessential spirit. In health research, wairua has become a proxy for ‘spiritual’, largely due to the Whare Tapa Whā model. Finding comfort in faith or spiritual beliefs was assessed using a single question from the FACIT-Sp (permission to use this item was granted by http://www.facit.org/FACITOrg), which had five response options ranging from ‘not at all’ to ‘very much’. For these analyses, responses to this question were grouped into two categories of ‘no’ and ‘little to very much’ [27].

##### Tinana (physical) dimension

Tinana can refer to ‘real’, ‘body’ and ‘in person’. Hence, in Te Whare Tapa Whā it is translated as the physical dimension in relational contrast to the metaphysical concept of wairua. The collection of pre-injury self-reported rating of overall health and chronic illness has been described above. However, for subsequent analyses, the prior chronic illness responses were grouped into those who reported ‘none’, ‘one’ or ‘two or more’ chronic illnesses. Injury severity derivation and groupings have also been described above.

##### Hinengaro (mental) dimension

Hinengaro refers to conscious thought, including intellect, cognisance and perception i.e. conscious subjective experience. Participants rated how things were overall for them before their injury, on a three-point scale (‘very happy’, ‘pretty happy’ or ‘not too happy’). Responses were grouped into two categories – ‘not too happy’ and ‘pretty/very happy’ for these analyses [28]. Self-efficacy was measured based on the General Self-Efficacy Scale, which assesses problem-solving capabilities relating to difficult demands in ten various aspects of life (such as solving difficult problems, accomplishing goals, and dealing with unexpected events) [29]. Response categories were ‘strongly disagree’, ‘disagree’, ‘neutral/mixed’, ‘agree’ and ‘strongly agree’ and were scored 0–4, respectively, which were grouped into ‘poor’ and ‘not poor’ (poor self-efficacy was defined as a summed score of ≤25 out of a maximum of 40). Participants were also asked about pre-injury depressive-type episodes (using DSM-III screening questions), i.e. if they had felt sad, blue or depressed; felt moody and irritable; or lost interest in things like work, hobbies or things they usually like to do for fun, for a period of two or more weeks in the 12 months before injury. If participants responded affirmatively they were categorised as ‘yes’ for ‘pre-injury depressive-type episode’ [30].

##### Socio-demographic characteristics

The collection of age, gender, working for pay and highest educational qualification data has been described earlier in this paper. Adequacy of pre-injury household income was collected by asking whether participants had ‘not enough’, ‘just enough’, ‘enough’ or ‘more than enough’ total household income to meet their everyday needs [21]. For these analyses, the last three responses were grouped together and compared with those who said they had ‘not enough’.

### Statistical analysis

Univariate analyses were produced to understand the characteristics of the study sample, and then chi-square tests assessed the strengths of bivariate associations between life satisfaction following injury and the explanatory variables of interest.

A multivariate Poisson regression model, with robust standard errors [31], was built using a stepwise backward selection procedure with a threshold p-value of 0.15 to explore the relationships between potential explanatory variables and life satisfaction three months after injury. Instead of estimating odds ratios for binary outcomes, this type of model allows direct estimation of relative risks. All explanatory variables in the univariate analyses in were included in this model as potential predictors. Age, gender, NISS and time from injury to interview were forced into the model so that the resultant estimates were adjusted for these four variables. The resultant model was compared to the original model using Akaike Information Criteria (AIC) for goodness of fit [32]. Participants with non-missing information for all applicable variables were included in the model. Consequently, the multivariate results presented are based on data from 535 participants.

Stata 12.1 was used for the analyses in this paper [33].

## Results

A range of baseline pre-injury characteristic data for the Māori cohort (n = 566; 20% of the total POIS cohort) is presented in Table 1. The median time to first interview for this Māori cohort was 3.1 months post-injury (inter-quartile range of 2.5-4.1 months).

**Table 1:** **Descriptive characteristics of Māori POIS participants** (**n** = **566**)

**Characteristic**		**N**	**%**
**Gender**			
	Male	371	65.5
	Female	195	34.5
**Age (at interview 1 in years)**			
	18-24	96	17
	25-34	135	23.9
	35-44	135	23.9
	45-54	138	24.4
	55-64	62	11
**Ethnicity**			
	Sole Māori	275	48.6
	Māori and other ethnicities	291	51.4
**Iwi known**			
	Yes	493	87.1
	No	59	10.4
	Missing/Don't know	14	2.5
**Region living (at interview 1)**			
	Auckland	145	25.6
	Manukau City	202	35.7
	Gisborne	108	19.1
	Otago	60	10.6
	Southland	51	9
**General health**			
	Excellent/Very good/Good	519	91.7
	Fair/Poor	45	8
Chronic illness*			
	Heart disease	33	5.8
	Stroke	12	2.1
	Diabetes	33	5.8
	Asthma	97	17.1
	Chronic bronchitis	13	2.3
	Arthritis	58	10.2
	Neck/back disorder	75	13.3
	Migraine	58	10.2
	Irritable bowel	15	2.7
	Depression	40	7.1
	Anxiety	29	5.1
	Other illnesses**	36	6.4
	None of the above	281	49.6
**Paid employment**			
	Not in paid employment	45	8
	Part-time (<30hrs/week)	38	6.7
	Full-time (≥30 hrs/week)	483	85.3
**Highest educational qualification**			
	No qualifications	147	26
	Secondary school	139	24.6
	Post-secondary school	270	47.7
**Personal income**			
	≤$15,000	34	6.0
	$15,001-$$30,000	75	13.3
	$30,001-$50,000	215	38.0
	$50,001-$70,000	91	16.1
	≥$70,001	54	9.5
	Undisclosed	97	17.1
Injury severity***			
	NISS 1-3	241	42.6
	NISS 4-6	220	38.9
	NISS >6	82	14.5

*Multiple illnesses allowed for each participant.

**Includes osteoporosis, chronic obstructive pulmonary disease, cancer, epilepsy, stomach ulcers, ME (chronic fatigue syndrome), bipolar disorder, schizophrenia, multiple sclerosis, motor neurone disease.

***Injury-related variable. All other variables are pre-injury.

Note: Column totals for each variable may vary and do not necessarily add to 566 as those with missing values have not been reported.

Most (66%) of the POIS Māori cohort is male. The mean age at first interview was 38.8 years (SD = 12.4 years). All those who identified at least one ethnicity as Māori are included in our Māori cohort. Of these, 49% identified Māori as their sole ethnicity. The majority (87%) reported that they knew their iwi (tribe). The majority were living in either Manukau City or Auckland (two cities within the largest metropolitan area of the country) at the time of injury. The majority (92%) reported having good to excellent overall general health pre-injury. One or more pre-injury comorbidities were reported by 285 people (50%), with asthma being the most prevalent (17%) followed by neck/back disorders (13%). The majority (92%) were in either full- or part-time paid employment. Just over a quarter reported having no secondary school qualifications and 48% reported a post-secondary school qualification. Of the 83% who reported their personal income before injury, the mean income in the year before injury was $47,247 and the median $41,000 (in New Zealand dollars).

Three participants had missing responses to the post-injury life satisfaction outcome question and were therefore excluded from subsequent analyses in this paper. Of the remaining 563 participants, 402 (71%) reported they were satisfied with life as a whole three months after injury, compared to 93% reporting they were satisfied with life pre-injury.

Table 2 shows some univariate relationships between variables of interest, grouped according to Te Whare Tapa Whā dimensions and socio-demographic characteristics, and life satisfaction outcome three months after injury. Within the whānau dimension, 94% of the cohort was satisfied with their pre-injury global social relationships, yet only 73% of this group reported satisfaction with life post-injury. Within the hinengaro dimension, the majority of the cohort was happy with things overall pre-injury and again 73% of this group reported satisfaction with life post-injury. Just over half of those who had poor self-efficacy pre-injury were satisfied with life post-injury, while of the 90% with ‘not poor’ pre-injury self-efficacy, 73% reported satisfaction with life. Likewise, those who experienced a pre-injury depressive-type episode were less likely to report life satisfaction post-injury, although 64% of this group did report satisfaction with life post-injury. There was a lack of statistical significance with other variables in the various dimensions (including in the socio-demographic grouping).

**Table 2:** **Te Whare Tapa Whā and socio**-**demographic characteristics and life satisfaction for Māori soon after injury**

				**Satisfied with life**		
**Whare Tapa Whā Dimension**	**Variable**		**Total**	n*	**%**	**P value**
**Whānau**	Global social relationships					
		Satisfied	528	387	73	<0.01
		Not satisfied	34	14	41	
	Family involvement					
		Very large/Large	483	352	73	0.10
		Small/Very small	77	49	64	
**Wairua**	Comfort in faith and spiritual beliefs					
		No	136	98	72	0.79
		Little to Very much	408	289	71	
**Tinana**	General health					
		Good to Excellent	517	369	71	0.90
		Fair/Poor	44	31	70	
	Prior chronic illness					
		0	281	206	73	0.38
		1	149	104	70	
		2+	120	80	67	
	Injury severity**					
		NISS 1-3	238	180	76	0.09
		NISS 4-6	220	154	70	
		NISS >6	82	52	63	
**Hinengaro**	Overall happiness					
		Pretty happy/Very happy	542	394	73	<0.01
		Not too happy	21	8	38	
	General self-efficacy					
		Not poor	508	371	73	0.004
		Poor**	52	28	54	
	Depressive-type episode					
		No	391	293	75	0.01
		Yes	171	109	64	
**Socio**-**demographic**	Paid employment					
		Full-time (≥30hrs/week)	481	346	72	0.48
		Part-time (<30hrs/week)	38	28	74	
		Not in paid employment	44	28	64	
	Highest educational qualification					
		No qualifications	146	105	72	0.53
		Secondary school	269	187	70	
		Post-secondary school	139	104	75	
	Adequacy of household income					
		Not Enough	66	41	62	0.08
		Just/Enough/More than	494	359	73	

*Row percentage.

**Injury-related variable. All other variables are pre-injury.

Note: Column totals for each variable may vary and do not necessarily add to 563 as those with missing values have not been reported.

Table 3 presents the multivariable model for life satisfaction. Those participants with an injury severity NISS >6 were 20% less likely to be satisfied with life three months after injury (RR = 0.80, 95% CI: 0.67-0.96) compared to those who had severities of NISS 1–3. Those not satisfied with pre-injury social relationships were 49% less likely to be satisfied with life three months after injury (RR = 0.51, 95% CI: 0.32-0.82). Those with poor general self-efficacy pre-injury were also less likely to be satisfied with life three months after injury (RR = 0.68, 95% CI: 0.51-0.92) compared to those whose self-efficacy was not poor.

**Table 3:** Multivariate analysis for life satisfaction for Māori three months after injury

**Variable**		**RR**	**95% CI**		**P value**
**Age at (interview 1 in years)**					
	18-24	Referent			0.72
	25-34	1.04	0.88	1.22	
	35-44	0.97	0.82	1.16	
	45-54	0.96	0.81	1.15	
	55-64	1.08	0.89	1.31	
**Gender**					
	Male	Referent			
	Female	0.93	0.82	1.04	0.21
**Injury severity (NISS)**					
	1-3	Referent			0.05
	4-6	0.91	0.81	1.02	
	>6	0.80	0.67	0.96	
**Pre**-**injury global social relationships**					
	Satisfied	Referent			
	Not satisfied	0.51	0.32	0.82	0.01
**Pre**-**injury general self**-**efficacy**					
	Not poor	Referent			
	Poor	0.68	0.51	0.92	0.01

No statistically significant associations between age or gender and life satisfaction three months after injury were observed.

## Discussion

Since this is the first publication that specifically analyses the Māori POIS data, we have presented a wide range of pre-injury descriptive characteristics. The majority of the cohort was male. This was expected due to the greater proportion of males on the ACC entitlement claims’ register, from which participants were recruited. The mean age at first interview for this cohort was 38.8 years. This is younger than that of the total POIS cohort (mean age = 41.4 years) but reflects the population distribution in New Zealand where the median age of the Māori population is 13.2 years younger than that of the total population [34]. Almost half of this cohort reported Māori ethnicity as their sole ethnicity. This pattern is similar to that observed in the New Zealand Census [35]. Despite half of the cohort reporting one or more pre-injury chronic illnesses, the overwhelming majority (92%) also reported having ‘good’ to ‘excellent’ overall pre-injury health. According to NISS, 81.4% of the cohort has ‘least severe’ or ‘middle severity’ injuries. One of the strengths of POIS is that we have a range of injury types and severities in the study cohort. Many injury outcome studies tend to focus on specific injury types (e.g., spinal cord injury or traumatic brain injury), causes (e.g., motor vehicle traffic crashes of falls) or severities (e.g. emergency department patients).

From our analyses, just over 20% of the cohort were less satisfied with life three months after injury than before (i.e., 71% three months after injury compared to 93% pre-injury). Despite the great majority of Māori reporting being satisfied with their life three months after injury, nearly a third were not (29%); this suggests that such research investigating outcomes and reasons for good (and poor) outcomes is warranted.

Variables from all four Te Whare Tapa Whā dimensions were included for consideration in the building of the multivariable model. Yet only having a more severe injury (according to NISS; tinana dimension), not being satisfied with pre-injury social relationships (such as the quality and frequency of relationships and contact with their partner, relatives and friends; whānau dimension), and having poor self-efficacy (such as solving difficult problems, accomplishing goals, and dealing with unexpected events; hinengaro dimension) were independently associated with being less likely to be satisfied with life three months after injury.

Interestingly, the variable that we had grouped into the wairua dimension (i.e., comfort in faith and spiritual beliefs) was not retained in the model. Additionally, overall happiness and health pre-injury were not independently associated with life satisfaction at three months after injury. Furthermore, despite identifying additional socio-demographic characteristics as potential predictors of post-injury life satisfaction (such as adequacy of pre-injury household income), none of these were found to be independently associated with the outcome of interest.

One of the obvious limitations of the present analyses is that we re-interpreted a questionnaire originally designed for the general population via an indigenous health model. As discussed previously, POIS was not set up to specifically measure aspects of these dimensions, although the majority of the questions in the first interview were able to be grouped into the four dimensions. Regardless, there remains definitional incongruence based on epistemological differences that limits the results. For example, the wairua (spiritual) dimension can encompass various notions of ‘faith’ and ‘spiritual’ beliefs, but more readily accounts for an entity’s spiritual essence. Interestingly, this was also highlighted as a potential limitation of POIS in Delaibatiki-Cammock *et al*.’s paper [36] with regard to POIS’s ability to capture Pacific health values identified by the Fonofale model [37]. Therefore, future studies wanting to investigate ‘spirituality’ aspects in greater detail should carefully consider such potential definitional differences during questionnaire development.

It is also important to remember that these results are from a sample of Māori who have gained access to the ACC. We are therefore unable to extrapolate our findings to those not accessing ACC. We are very aware that people not accessing ACC support may have very different experiences after injury and further research investigating these is required.

Our results indicate that for health providers and agencies seeking to help improve life satisfaction among Māori following injury, perhaps greater effort should be put into identifying, and then working alongside, injured Māori who reported poor self-efficacy, were not satisfied with their pre-injury social relationships, and who had ‘severe’ injuries to ensure greater likelihood of satisfaction with life in the early stages after injury.

To our knowledge, this study is one of very few that have used the commonly referenced Te Whare Tapa Whā model as a framework for informing quantitative analysis of a Māori cohort. This article does not aim to ‘test’ the model, or its ability to predict post-injury outcomes for Māori. Rather, it has been used as a framework to help inform the injury outcome of interest (i.e. post-injury life satisfaction) and its potentially important predictors.

As discussed previously, there is very little published literature that examines injury outcomes for Māori. This paper, and subsequent others from POIS, will help address the current knowledge gap in this important area. There is also very little published literature internationally that explores outcomes after injury for other indigenous populations. By focusing on predictors for specific outcomes for Māori soon after injury in POIS, we hope that this will encourage researchers to do so for other indigenous populations.

## Conclusions

It is encouraging that for Māori gaining access to the ACC scheme, the majority are satisfied with their life as a whole three months after injury. Nevertheless, a third of the participants were not. Our findings indicate that, despite the study’s limitations, a greater focus during the early days after injury on those who have severe injuries, were not satisfied with pre-injury social relationships and had poor self-efficacy pre-injury may help to improve life satisfaction soon after injury for Māori. This paper also provides a unique, important and relevant interpretation of results for Māori via a health model underpinned by indigenous concepts.

## Competing interests

The authors declare that they have no competing interests.

## Authors’ contributions

EW, SD and BH jointly planned the scope and content of the paper. AS undertook the statistical analyses. EW, SD, BH and AS were actively involved in interpreting the results and contributing to various drafts of the manuscript. All authors read and approved the final manuscript.

## References

[B1] Ajwani S Blakely T Robson B Tobias M Bonne M Decades of Disparity: Ethnic Mortality Trends in New Zealand 1980–1999 2003 Wellington, New Zealand: Ministry of Health 15475978

[B2] Ministry of Health, University of Otago Decades of Disparity III: Ethnic and Socioeconomic Inequalities in Mortality, New Zealand 1981–1999 2006 Wellington, New Zealand: Ministry of Health

[B3] Reid P Robson B Robson B, Harris R Understanding Health Inequities Hauora: Māori Standards of Health IV. A study of the years 2000–2005 2007 Wellington, New Zealand: Te Rōpū Rangahau Hauora a Eru Pōmare 3 10

[B4] Ministry of Health Tatau Kahukura: Māori Health Chart Book 2010, 2nd Edition 2010 Wellington, New Zealand: Ministry of Health

[B5] Office for Disability Issues, Statistics New Zealand Disability and Māori in New Zealand in 2006: Results from the New Zealand Disability Survey 2010 Wellington, New Zealand: Statistics New Zealand

[B6] Langley J Broughton J Nga Tatauranga: Injury to Māori 1998 Dunedin, New Zealand: Injury Prevention Research Unit and the Ngāi Tahu Māori Health Research Unit (University of Otago)

[B7] Broughton J Langley J Injury to Maori. II: serious injury N Z Med J 2000 113 511 513 11198512

[B8] Langley J Broughton J Injury to Maori. I: fatalities N Z Med J 2000 113 508 510 11198511

[B9] Creamer G Civil I Ng A Adams D Cacala S Koelmeyer T Thompson J Ethnicity of severe trauma patients: results of a population-based study, Auckland, New Zealand 2004 N Z Med J 2010 123 26 32 20648097

[B10] Sargent M Begg D Broughton J Stephenson S Wright C Baxter J Motor vehicle traffic crashes involving Maori N Z Med J 2004 117 U746 14999305

[B11] Marrone S Understanding barriers to health care: a review of disparities in health care services among indigenous populations Int J Circumpolar Health 2007 66 188 198 17655060 10.3402/ijch.v66i3.18254

[B12] Mauri Ora Associates Māori Experience of ACC: Mauri Ora Associates Final Report for Department of Labour 2010 Auckland: Mauri Ora Associates

[B13] Derrett S Davie G Ameratunga S Wyeth E Colhoun S Wilson S Samaranayaka A Lilley R Hokowhitu B Hansen P Langley J Prospective Outcomes of Injury Study: recruitment, and participant characteristics, health and disability status Inj Prev 2011 17 415 418 21724742 10.1136/injuryprev-2011-040044

[B14] Derrett S Langley J Hokowhitu B Ameratunga S Hansen P Davie G Wyeth E Lilley R Prospective Outcomes of Injury Study Inj Prev 2009 15 e3 19805606 10.1136/ip.2009.022558a

[B15] Wyeth EH Derrett S Hokowhitu B Hall C Langley J Rangatiratanga and Ōritetanga: responses to the Treaty of Waitangi in a New Zealand study Ethn Health 2010 15 303 316 20461598 10.1080/13557851003721194

[B16] Smith LT Decolonizing Methodologies - Research and Indigenous Peoples. 6th impression 1999 Dunedin, New Zealand: University of Otago Press

[B17] Durie M Durie M Tirohanga Māori - Maori Health Perspectives Whaiora - Māori Health Development 1994 2 Melbourne, Australia: Oxford University Press 66 80

[B18] Durie MH A Maori Perspective of Health Soc Sci Med 1985 20 483 486 3992288 10.1016/0277-9536(85)90363-6

[B19] Making a Claim [http://www.acc.co.nz/making-a-claim/index.htm]

[B20] Statistics New Zealand New Zealand Census of Population and Dwellings - Individual Form 2006 Wellington, New Zealand: Statistics New Zealand

[B21] Ministry of Social Development Direct Measurement of Living Standards: The New Zealand ELSI Scale - Survey of Working Age People in 2000 2000 Wellington, New Zealand: Ministry of Social Development

[B22] Ware JE Snow KK Kosinski M Gandek B SF-36 Health Survey: Manual and Interpretation Guide 2000 Lincoln, RI: QualityMetric Inc.

[B23] Ministry of Health A Portrait of Health. Key Results of the 2006/07 New Zealand Health Survey 2008 Wellington, New Zealand: Ministry of Health

[B24] Stevenson M Segui-Gomez M Lescohier I Di Scala C McDonald-Smith G An overview of the injury severity score and the new injury severity score Inj Prev 2001 7 10 13 11289527 10.1136/ip.7.1.10 PMC1730702

[B25] Derrett S Samaranayaka A Wilson S Langley J Ameratunga S Cameron ID Lilley R Wyeth E Davie G Prevalence and Predictors of Sub-Acute Phase Disability after Injury among Hospitalised and Non-Hospitalised Groups: A Longitudinal Cohort Study PLoS One 2012 7 e44909 22984584 10.1371/journal.pone.0044909 PMC3439380

[B26] Fugl-Meyer AR Bränholm I-B Fugl-Meyer KS Happiness and Domain-specific Life Satisfcation in Adult Northern Swedes Clin Rehab 1991 5 25 33

[B27] Peterman AH Fitchett G Brady MJ Hernandez L Cella D Measuring spiritual well-being in people with cancer: the functional assessment of chronic illness therapy-Spiritual Well-being Scale (FACIT-Sp) Ann Behav Med 2002 24 49 58 12008794 10.1207/S15324796ABM2401_06

[B28] Uppal S Impact of the timing, type and severity of disability on the subjective well-being of individuals with disabilities Soc Sci Med 2006 63 525 539 16530905 10.1016/j.socscimed.2006.01.016

[B29] Schwarzer R Jerusalem M Weinman J, Wright S, Johnston M Generalized Self-Efficacy Scale Measures in Health Psychology: A User's Portfolio Causal and Control Beliefs 1995 Windsor, England: NFER-NELSON 35 37

[B30] American Psychiatric Association Committee of Nomenclature and Statistics Diagnostic and Statistical Manual of Mental Disorder-3rd Edition (DSM-3) 1980 Washington, DC: American Psychiatric Association

[B31] Zou G A modified poisson regression approach to prospective studies with binary data Am J Epidemiol 2004 159 702 706 15033648 10.1093/aje/kwh090

[B32] Sakamoto Y Ishiguro M Kitagawa G Akaike Information Criterion Statistics 1986 Dordrecht: Reidel Publishing Company

[B33] StataCorp Stata: Release 12. Statistical Software 2011 College Station, Texas: StataCorp LP

[B34] Statistics New Zealand QuickStats National Highlights (Revised edition) 2007 Wellington, New Zealand: Statistics New Zealand

[B35] Statistics New Zealand QuickStats About Māori (Revised edition) 2007 Wellington, New Zealand: Statistics New Zealand

[B36] Delaibatiki-Cammock R Derrett S Davie G Langley J Sopoaga F Injury to Pacific People in New Zealand: Pre-injury characteristics and early health outcomes - results from a cohort study Australas Epidemiol 2012 19 17 21

[B37] Fonofale Model of Health [http://www.hpforum.org.nz/resources/Fonofalemodel.pdf]

